# Looking for a broader mindset in psychometrics: the case for more participatory measurement practices

**DOI:** 10.3389/fpsyg.2024.1389640

**Published:** 2024-03-27

**Authors:** Javiera Paredes, David Carré

**Affiliations:** ^1^Laboratorio de Lenguaje, Interacción y Fenomenología, Escuela de Psicología, Pontificia Universidad Católica de Chile, Santiago, Chile; ^2^Instituto de Ciencias de la Salud, Universidad de O’Higgins, Rancagua, Chile

**Keywords:** psychometrics, measurement, psychology, participation, communities

## Abstract

Psychometrics and the consequences of its use as *the* method of quantitative empirical psychology has been continuously criticized by both psychologists and psychometrists. However, the scope of the possible solutions to these issues has been mostly focused on the establishment of methodological-statistical best practices for researchers, without any regard to the pitfalls of previous stages of measurement as well as theory development of the targeted phenomenon. Conversely, other researchers advance the idea that, since psychometrics is riddled with many issues, the best way forward is a complete rework of the discipline even if it leaves psychologists and other practitioners without any way to measure quantitatively for a long period of time. Given these tensions, we therefore advocate for an alternative path to consider while we work on making substantive change in measurement. We propose a set of research practices focusing on the inclusion and active participation of groups involved in measurement activities, such as psychometrists, researchers but most importantly practitioners and potential participants. Involving a wider community while measuring in psychology could tackle some key issues that would take us closer to a more authentic approach to our phenomenon of interest.

## Introduction

By looking at the current landscape of psychology, there are many reasons to argue that psychometrics is one of the most successful subfields of the discipline (Borsboom and Wijsen, 2017; Craig, 2017). It is cited and used by almost every empirical work published in recent decades (Jones and Thissen, 2006). Even more so, its measurement standards have become basic requisites asked by most scientific journals to even consider a manuscript for review (Eich, 2014; Trafimow and Marks, 2015). Accordingly, it has become a–if not *the*–core course of almost every undergraduate and graduate program in any field related to psychological science (Friedrich et al., 2000; TARG Meta-Research Group, 2022). In brief, contemporary psychological research seems to involve putting psychometrics into practice.

Considering its success and widespread influence, it is nothing short of paradoxical that psychometrics has been the target of the harshest critiques within and beyond the discipline during recent decades. The range of these critiques has gone from questioning whether the last 50 years of psychometric research has any value at all (Salzberger, 2013) to arguing that psychometrics does not actually do measurement–at least in the metrological sense of the term (Uher, 2021a,b). Thus, psychometrics has been criticized to its core, ultimately calling for its refoundation.

Even if we look past these fundamental critiques, we find that researchers within the psychometrics community have also raised a number of issues; which they have tried to address with varying degrees of success. Among these it is possible to find the replicability crisis (Stevens, 2017; Anvari and Lakens, 2018), all sorts of data dredging practices, commonly known as *p-hacking* (Szucs, 2016; Stefan and Schönbrodt, 2023), or the lack of pre-registering protocols (van 't Veer and Giner-Sorolla, 2016; Spitzer and Mueller, 2023). While the latter group of critiques has called for necessary improvements of standards and practices within psychometrics and psychology, they have not really addressed the breadth and depth of the criticisms made by other scholars (e.g., Salzberger, 2013; Uher, 2021b). Neither have they tried to: during the last decade most of the psychometric community have been devoted to developing procedures and practices aimed to prevent the misuse of psychometrics by researchers. Thus, the effort to solve the aforementioned issues has focused on turning detailed data-handling protocols and replication studies into common practices within psychological research. But they do not question—with exceptions (e.g., Bauer, 2024)–whether psychometrics actually measures what it aims to measure or even if it measures something at all. This second group of critiques thus follows a line of renovating psychometrics rather than rebuilding it. This, in turn, makes the dialog between both camps unlikely: as one side aims to change (almost) everything from the ground up while the other looks to correct and prevent malpractices.

In this scenario, the present work neither aims to deepen the re-foundational critiques that have been posed on psychometrics, nor proposes adjustments to current measurement practices hoping to solve all the ailments of the discipline. Instead, we aim to build upon already identified issues to propose alternative *research* practices for psychometrics that broaden the mindset of this sub discipline. We argue that these practices could contribute in closing the gap between existing critiques and the current measurement standards in a feasible way.

We do so for two reasons. First, despite the recognition of its many shortcomings and the conceptual critiques against its tenets and practices, psychometrics keeps—and probably will keep—being utilized by practitioners and researchers alike due to its standing and usefulness. Thus, the prospect of rebuilding the discipline, starting something new based upon completely different tenets, seems simply unfeasible. Second, because we do acknowledge that changes in psychometric practices have to go beyond pre-registering, statistical and open data practices. In order to make changes to psychometrics substantial, they have to alter the direction in which current research and measurement practices are pointed. This is why we consider that more transparency, expressed through different procedures (e.g., Hardwicke and Vazire, 2023), is not enough by itself to make psychometrics–and its impact over psychological research and practice at large–overcome its fundamental challenges.

For these reasons, what we deem essential is a change in the mindset of psychometrics toward a broader one. A change that does not aim to make psychometrics renounce to technical and mathematical standards (which would be an oxymoron), but not to make these standards its only interest and ultimate goal. We are not alone in proposing a change of this kind. In a recent editorial, the outgoing editor-in-chief of *Psychological Science*–one of the journals with highest impact factor in psychology–calls for a similar change: to stop focusing all the attention on methodological, procedural issues and start thinking about how psychological research actually speaks about the phenomena of interest, which she aptly terms as authenticity (Bauer, 2024). We share with Bauer (2024) that doing *more* is not enough, it has to be done *differently*.

In the following we argue in favor of a set of practices that could–and should–be done differently: participatory processes within measurement practices. More specifically, we focus on the role that promoting participation could have on achieving a better understanding of the measurement processes involved in the most common psychometric instruments–namely, questionnaires (see Tourangeau et al., 2000). As it has been proposed (Uher, 2021b), the person being the instrument of measurement is one of the essential shortcomings of psychometrics. We consider that, for a discipline devoted to human-driven measurement, this is rather one of the essential challenges of psychometrics.

## Humans as data generation instruments

One of the fundamental issues identified by critics of psychometrics focuses on the human-based nature of measurement in psychology (Uher, 2021a,b). Since the use of surveys in psychology is extensive, the participant–as defined by metrology–is regarded as the source of the quantitative data. It is the person who reads, understands and interprets the instrument the one to give an answer related to the construct that the survey ultimately refers to (Uher, 2021b). Different to this response process is the structure of the scale itself. Scales may or may not follow different psychometric standards, which is determined by the statistical analysis of the numerical responses that were provided by human action.

The metrological perspective, however, is in clear opposition to what psychology typically considers as the source of data generated by quantitative instruments. In the common use of psychometrics by psychologists and practitioners, the measurement instrument is determined by the number of questionnaire items defined as latent representatives of the studied phenomena. The participants who respond to the survey are not usually considered primary players in the response process beyond providing data for validation processes during measurement development (Hughes, 2018; Levac et al., 2019; Reynolds et al., 2021). Therefore, after validation, instruments seem to gain a life of its own that transcends the way in which respondents interact with them.

This naïve approach to quantitative measurement involving instruments such as surveys in psychology implies a double source of possible error. Participants, according to this view, produce an answer to the latent construct that the survey asks for. But the former neglects that the construction of the items already has an identified source of error, which stems from the distance between the particular construct proposed by instrument-developers and the theoretical definition of the psychological concept that encompasses all its possible modes of presentation (Uher, 2018). This first source of error, namely the distance between the construct and its theoretical definition, has been long identified by psychologists through the empirical testing of their measurement models. Researchers have long discussed the inability of quantitative psychological models to achieve complete fidelity to the phenomena studied through the developed measurement instruments (Oberauer and Lewandowsky, 2019; Eronen and Bringmann, 2021).

The second possible source of error emerges every time that a particular participant answers each item of the survey. How do we know that the cognitive and interpretative process is the same in every person that approaches the instrument? This well-known issue is commonly addressed in the process of developing measures through tools like the cognitive interview. This interview aims to figure out the response processes to make sure that each item is understood as the researchers intended it to (Tourangeau et al., 2000). This approach, however, does not solve the fact that each singular process of response could bring very different outcomes by the only act of interpretation of each participant. For example, how does a headache affect the process of understanding what happiness is? Contextual elements, beyond the cumulative of cognitive representational contents assigned to each definition of an item during validation, could be an inextricable source of error related to the human-based nature of measurement in psychology.

To summarize, the measurement process in psychology relies on two different user-dependent activities: one that involves the appropriate understanding of the scale functioning by researchers and practitioners; and the agreement of each person on the definition of the phenomena presented as items in the questionnaires. It is in this regard that person-centered interactions and instruments are considered by metrologists as one the roots of measurement errors in psychological assessment. Numerical traceability is one of the critical aims in quantitative measurement to ensure a successful data generation process. Successful, in this context, implies the existence of a clear link between the numerical attributes assigned to psychological phenomena and certain pre-established standards. For a link that directly relates the numerical attribute with the psychological phenomena is the only way to make results obtained from questionnaires to be non-dependent on the users of the instrument (Uher, 2021b). Therefore, when we consider the human-based nature of measurement described above, numerical traceability in psychology is not achievable.

The recommendation of experts when confronted with the issue of the lack of numerical traceability in psychology has been to search for practices to ensure the establishment of clear and distinct *intersubjective* meanings of the numerical results of each item (e.g., Hughes, 2018; Reynolds et al., 2021). A successful example of these practices is identified in the development of cognitive abilities instruments such as the Wechsler Adult Intelligence Scale (WAIS) (Benson et al., 2010; Weiss et al., 2010). Since the process of cognitive evaluation has an additional human-based source of error (i.e., the test applicator) and the stakes involved in this kind of assessment process are high, the need of establishing clear meanings regarding numerical results is just as key as the conceptual nature of the constructs evaluated. The results of these practices are certainly satisfactory, as the meaning of numerical results of the WAIS are fairly standard and unambiguous within the cognitive assessment community.

Here it is important to note that we see no contradiction between, on the one hand, improving measurement practices in order to provide an account of the phenomena that is closer to the theoretical grounds proposed and, on the other, advancing toward more precise theoretical structures that allow numerical traceability in psychology. Therefore, we follow the experts’ recommendation and further argue that there is much to be gained in attempting to make conventional, intersubjective agreements about numerical results more common across the discipline; for examples like the one described above are the exception rather than the norm. To do so, as we develop in the following, it is essential to involve actors beyond psychometrics to make such intersubjective agreements actually agreements and not yet another technical recommendation.

## Participatory processes as a cornerstone of psychological measurement

As we argued at the beginning of this work, we consider that psychometrics is in dire need of broadening its mindset. By this we mean that rather than trying to do more—or less—of what is currently done, different things should be done instead. Thinking along these lines, we are in favor of promoting community participatory processes as a pivotal element of measurement practices in psychology. By community participatory processes we are standing for the inclusion of researchers, practitioners and users of psychological instruments.

As noted above, the inclusion of best practices in psychological research and publication has been the cornerstone of the attempts to solve the issues regarding measurement in psychology (e.g., Flake and Fried, 2020; Aguinis et al., 2021). Naturally, the community involved in these changes has mostly included psychometrists and researchers in psychology. We believe, however, that the efforts toward improving measurement instruments should also involve the voices of more practitioners and everyday users of these instruments, even–or especially–if they are not trained in psychological science.

Practitioners and users of the instruments developed by psychometrists and researchers are essential stakeholders that possess insights into some pressing issues in this discussion, like numeric traceability. Achieving agreement about the intersubjective meaning of scale items is one example, as described above. An accurate analysis of these problems only can be conducted when the developers of the instruments can account for the understanding of all the people involved in these practices. Users and practitioners, therefore, should not only be eventually included in the process in the final stages of development (i.e., validation) but also in previous steps, thus assisting the construction of measurements that are sensible to the phenomena of interest.

Respondents, on the other hand, are a source of crucial information regarding the actual interpretation and response processes in surveys. While we may rely on the expertise of psychologists, psychometrists and, sometimes, the teams that apply these instruments, it is not enough to capture the real meaning given by people to each item. And the main issue still remains intact if we consider that we as psychologists still rely heavily on samples that do not necessarily represent the people who answer our surveys. WEIRD (western, educated, industrialized, rich, and democratic) or Mechanical Turk samples have been the focus of past and current academic discussion regarding their suitability as a source of data in psychological research (Keith and Harms, 2016; Webb and Tangney, 2022).

A fair counterpoint to more participatory practices is the issue of viability. The inclusion of every single prospective practitioner or user, and including each meaning considered to the item construction and instrument it is simply not achievable, especially when means are scarce and time is limited. But that would be taking the argument to an unreasonable extreme. What we are proposing here is making efforts for a wider and more nuanced understanding of how different people, communities and cultures approach and answer the scales that are developed. Participation is anything but binary, thus we are calling for advancing toward more inclusion of different actors and not for a strict process of co-creation.

Once again, the way in which cognitive assessment has included participatory practices offers valuable insights. Even without modifying the instruments used, this area has shown how to improve existent measurement practices in psychology. Due to the practical impact that such an assessment has, it commonly involves lengthy validation efforts that ensure that the data generation instruments–namely, people–are participating and responding in such a way that can be compared to other persons in other areas of the world. But the stakes of psychological measurement certainly go beyond cognitive assessment. Determining levels of prejudice among members of a community; assessing whether a person meets a specific personality profile; establishing the impact of an intervention in the improvement of memory. These examples, as many others do, remind us of the stakes involved in developing psychometric instruments. They should also push us to make every possible effort to improve measurement practices–even if it involves costlier and slower development processes that include participation.

## The siren’s call for quick data collection

In this perspective work we have argued in favor of expanding the current mindset of psychometrics in order to look beyond technical and statistical concerns. We do so to advance potential solutions to the pressing challenges of the subdiscipline without waiting for its refoundation or hoping for minor renovations. Although a complex endeavor, we cannot ignore precisely what makes psychological measurement prone to error, the human-based nature of the data-generation instrument.

Instead of trying to look past this human nature through sophisticated means, we have proposed ways to understand this nature better through participatory practices. Therefore, the psychometric and psychological communities of researchers should not disregard the attitudes, meanings and knowledge of other groups involved in measurement–that is if they want to develop instruments that account for the complex psychological phenomena they measure.

These ideas, moreover, could also be applied to measurement in other disciplines in which participation has not been a priority. In educational assessment, a number of works have emphasized participation mostly through self- and peer-assessment practices (e.g., Li et al., 2016) and teacher’s practices for communicating their assessment expectations (e.g., Stefani, 1998). In standardized testing, the general absence of participatory practices should not come as a surprise considering that the *Standards for Educational And Psychological Testing* (American Educational Research Association, American Psychological Association, and National Council on Measurement in Education, 2014) mentions ‘participatory’ only once in its 130 pages. Therefore, participation has been reduced to processes that do not actually involve students on what or how their learning is assessed (Aarskog, 2021). In health sciences, on the other hand, there is devoted effort to enhance user’s participation in multiple dimensions of healthcare (Angel and Frederiksen, 2015); except in the development of instruments used to assess health outputs. In sum, we envision a significant space for including the practices processes we propose, although the specific way in which different fields could bring these ideas into everyday practice, however, remains an open discussion that we hope to trigger with this work.

We have no doubts that our position does not sit well with many researchers in psychometrics who honestly hope to address every single issue through technical means. To them, we can only repeat the blunt conclusion of Patricia Bauer’s recent editorial piece: “(...) we must resist the siren’s call for quick data collection, with instruments that barely scratch the surface of a complex psychological construct, and that offer sweeping conclusions seemingly without limits on their generalizability.” (2024, p.3) One of the ways in which we can resist that call is bringing more voices into the work of psychometrics and make them participate in the development of psychological measurement.

## Data availability statement

The original contributions presented in the study are included in the article/supplementary material, further inquiries can be directed to the corresponding author.

## Author contributions

JP: Conceptualization, Writing – original draft, Writing – review & editing. DC: Conceptualization, Writing – original draft, Writing – review & editing.
